# A development study and randomised feasibility trial of a tailored intervention to improve activity and reduce falls in older adults with mild cognitive impairment and mild dementia

**DOI:** 10.1186/s40814-018-0239-y

**Published:** 2018-02-17

**Authors:** Rowan H. Harwood, Veronika van der Wardt, Sarah E. Goldberg, Fiona Kearney, Pip Logan, Vicky Hood-Moore, Vicky Booth, Jennie E. Hancox, Tahir Masud, Zoe Hoare, Andrew Brand, Rhiannon Tudor Edwards, Carys Jones, Roshan das Nair, Kristian Pollock, Maureen Godfrey, John R. F. Gladman, Kavita Vedhara, Helen Smith, Martin Orrell

**Affiliations:** 1 Health Care of Older People, Nottingham University Hospitals NHS Trust, Queens Medical Centre, Nottingham, NG7 2UH UK; 2 Division of Rehabilitation and Ageing, University of Nottingham, Nottingham, NG7 2UH UK; 3 School of Health Sciences, University of Nottingham, Nottingham, NG7 2UH UK; 4 NWORTH Clinical Trials Unit, Bangor University, Bangor, LL57 2PZ UK; 5 Centre for Health Economics and Medicines Evaluation, Bangor University, Bangor, LL57 2PZ UK; 6 Institute of Mental Health, University of Nottingham, Nottingham, NG8 1BB UK; 7 Division of Primary Care, University of Nottingham, Nottingham, NG7 2RD UK; 8 Mental Health Services for Older People, Nottinghamshire Healthcare NHS Foundation Trust, Highbury Hospital, Nottingham, NG6 9RD UK

**Keywords:** Dementia, Activities of daily living, Therapeutic exercise, Occupational therapy, Physiotherapy, Falls, Randomised controlled trial, Adherence, Economic evaluation, Process evaluation

## Abstract

**Background:**

People with dementia progressively lose abilities and are prone to falling. Exercise- and activity-based interventions hold the prospect of increasing abilities, reducing falls, and slowing decline in cognition. Current falls prevention approaches are poorly suited to people with dementia, however, and are of uncertain effectiveness. We used multiple sources, and a co-production approach, to develop a new intervention, which we will evaluate in a feasibility randomised controlled trial (RCT), with embedded adherence, process and economic analyses.

**Methods:**

We will recruit people with mild cognitive impairment or mild dementia from memory assessment clinics, and a family member or carer. We will randomise participants between a therapy programme with high intensity supervision over 12 months, a therapy programme with moderate intensity supervision over 3 months, and brief falls assessment and advice as a control intervention. The therapy programmes will be delivered at home by mental health specialist therapists and therapy assistants. We will measure activities of daily living, falls and a battery of intermediate and distal health status outcomes, including activity, balance, cognition, mood and quality of life. The main aim is to test recruitment and retention, intervention delivery, data collection and other trial processes in advance of a planned definitive RCT. We will also study motivation and adherence, and conduct a process evaluation to help understand why results occurred using mixed methods, including a qualitative interview study and scales measuring psychological, motivation and communication variables. We will undertake an economic study, including modelling of future impact and cost to end-of-life, and a social return on investment analysis.

**Discussion:**

In this study, we aim to better understand the practicalities of both intervention and research delivery, and to generate substantial new knowledge on motivation, adherence and the approach to economic analysis. This will enable us to refine a novel intervention to promote activity and safety after a diagnosis of dementia, which will be evaluated in a definitive randomised controlled trial.

**Trial registration:**

ClinicalTrials.gov: NCT02874300; ISRCTN 10550694.

## Background

Dementia is a syndrome of progressive and usually irreversible loss of memory and other cognitive functions including agnosia, apraxia, language and executive function, caused by a variety of brain diseases, and severe enough to interfere with daily function. Mild cognitive impairment (MCI) is defined by measureable memory loss or other cognitive decline in the absence of interference with daily function, but which progresses to dementia in about half of cases. Dementia affects 1% of those at age 65, 20% at age 80 and 30% or more at age 90. Prevalence in the UK is about 850,000, and expected to double by 2030 [1].

There is no cure for dementia, but acetylcholinesterase inhibitor drugs and cognitive stimulation therapy can improve cognition by a modest amount [2, 3]. There are few other interventions to maintain or improve health status. Two potential mechanisms for avoidable deterioration in cognition and function are crises associated with physical health problems (such as falls and their consequences), and progressive restriction of activity by the person with dementia or those who support and care for them, often with the goal of maintaining safety.

A fall is defined as unintentionally coming to rest on the floor or at a lower level, through whatever cause [4]. People with dementia and MCI are at high risk of falling with at least a twofold increased risk compared with cognitively normal older people [5–7]. This equates to an annual fall incidence of 60–80% [8–11]. Consequences of falls include fractures, other injuries, hospital attendance, the ‘fear of falling syndrome’, immobility and loss of independence. People with dementia also have a higher risk of fractures, hip fractures in particular, and poorer outcomes after fracture, compared with people who are cognitively intact [6, 12]. Up to a third of emergency hospital admissions occur in an older person with dementia, and over half of these are associated with a fall [13]. Each year, in the UK, there are 75,000 hip fractures, set to rise by half again in the next 10 years, and 200,000 other fragility fractures. The combined cost of these is over £2 billion per year, and use 1.6 million hospital bed days. Half of hip fractures occur in someone with dementia [14].

There has been extensive research into falls prevention in older people. Risk factors are muscle weakness, neurological disease, medications, poor vision and environmental factors. Multi-factorial interventions reduce risk [15, 16] but these interventions have not been shown to reduce falls in people with dementia or MCI [8, 17–19]. A systematic review of interventions concluded that they were poorly adapted to the needs of people with dementia [20]. Falls guidelines recommend that cognitive function is assessed, but do not say how to respond [21]. People with dementia have more ‘conventional’ falls risk factors than people of similar age without dementia [8]. They also have dementia-specific risk factors including: type and severity of dementia, specific cognitive and gait deficits, behavioural disturbances, and psychotropic drug use [11, 22, 23]. Studies highlight the importance of attention, and dual-task cost (increased risk when concentrating on two things at once) [24], manifestations of impaired executive function (ability to form, maintain, and shift mental set [25]). Abnormalities in executive function and gait are associated with falls [26, 27]. Dual-task and gait abnormalities are found early in dementia [11, 28–31] and MCI [32] beyond what would be considered ‘normal ageing’.

Potentially reversible risk factors provide opportunities to intervene before inevitable deterioration occurs. Systematic reviews have considered the impact of strength and balance training in older people, with and without dementia [19, 33–37]. Moderate-intensity exercise, 2–3 times a week, improves strength, gait speed, and ability in activity of daily living [38–42]. There may be additional benefit in slowing cognitive decline [38, 41, 43–47] although the size of this effect appears small. Training can improve executive function, dual-task performance and gait parameters [48–52]. Functionally orientated therapy can improve ability to perform activities of daily living [44, 53, 54]. There is insufficient evidence to confirm reduction in falls, improved mood or behaviour for people with dementia, or reduced carer strain [19, 20, 38, 40, 45, 55]. Customary levels of physical activity are low among older people [56].

The FINALEX trial of 12 months of twice-weekly, supervised exercise at home for people with established dementia and their co-resident spouse, reduced deterioration in activities of daily living and halved the rate of falling, from 3.1 to 1.4 falls per person-year [57]. Hospital admissions and overall costs were reduced. This demonstrates that intensive exercise is achievable, sustainable with the right support, and cost-effective. The challenge is how to achieve sufficient participation, adherence and persistence in the NHS and UK cultural environment, and to be inclusive, for example, of the 50% of people with early dementia who live alone.

A new intervention must lead to changes in individuals that are physiologically and neuro-psychologically credible, but must also take account of aspects of behaviour change [58–61]. The utility of current approaches to behaviour change (e.g. behaviour change wheel [58]) in the context of dementia is unknown, but provides a framework for further investigation. Barriers to sustaining a moderate to vigorous exercise programme include not perceiving oneself as being at risk from falls; perceived lack of relevance of exercise or other interventions; and focus on priorities such as maintaining independence, or family, social or domestic concerns [62–64]. Barriers to long-term adherence include forgetfulness, medical co-morbidities, planning problems and practical support. Motivational strategies might include supervision, tailoring, remote feedback, prompts, memory aids, goal setting, and rote-learning habit formation [65–72]. This problem has been studied in other conditions; for example, adherence to exercise in chronic musculo-skeletal pain [71], but overall evidence is sparse. It is not clear which, if any, strategies are acceptable and effective for people with dementia.

## Methods

### Aim

This research aims to develop and test a novel intervention to maintain activity and reduce falls in older adults with MCI and mild dementia.

The feasibility study aims to answer practicability and feasibility questions about recruitment and retention of participants, study procedures, delivery, intensity and burden of intervention, adherence, data collection and completion of outcomes measures, and implementation of a rehabilitation staff training programme to ensure that a planned large-scale trial is successful.

We will study how to maximise participant adherence with the programme, developing practical strategies to optimise adherence, and ascertaining in retrospect what did and did not work for particular individuals, both during the therapy programme and in the long term.

We will undertake a process evaluation, studying fidelity, understanding mechanisms and context, including barriers and facilitators to participation.

We will collect resource-use data to enable preliminary economic modelling.

### Study design

**Fig. 1:**
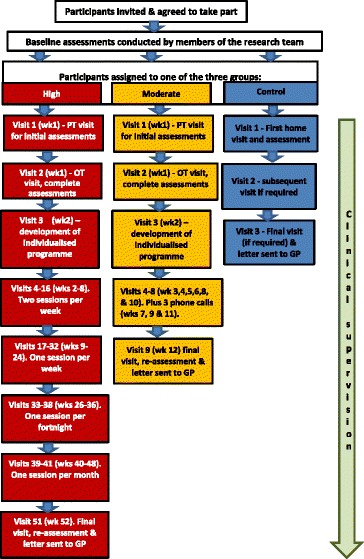
Overview of assessment and intervention. *PT* physiotherapist, *OT* occupational therapist, *GP* general practitioner/family doctor, *wk.* week

**Fig. 2:**
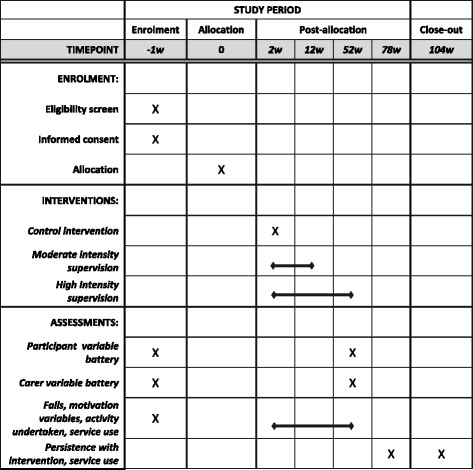
Schedule of enrolment, interventions and assessments

A two-centre, pragmatic, parallel group, feasibility RCT will be conducted (Figs. 1 and 2). After informed consent, participants will be randomised to one of two intervention arms (high- or moderate-intensity supervision) or a control group who will receive standard falls assessment and advice. Where possible, each participant will nominate a family member, friend or carer to be both an informant and a participant in their own right. A process evaluation [73], study of motivation and adherence, and a preliminary economic analysis will be undertaken, using mixed qualitative and quantitative methods.

### Setting

Recruitment will be from locality-based, secondary-care memory clinics, and therapy delivered in the participant’s home.

### Participants (inclusion, exclusion, withdrawal)

Eligible patient participants will be aged over 65 years and will have a diagnosis of mild dementia or MCI (of any subtype), attendance at a memory assessment service, or on the ‘Join Dementia Research’ register (a national initiative to encourage participation in dementia research [74]) with a cognitive score ranging 15–25 on the Montreal Cognitive Assessment (MoCA) [75], 18–26 on the standardised Mini-Mental State Examination (sMMSE) [76], or 60–90 on the Addenbrooke’s Cognitive Examination (ACE-III) [77].

Patient participants must be able to walk without human help, able to communicate in English, and to be able to see, hear and have dexterity sufficient to perform neuropsychological tests. Patient participants must have capacity to give consent to participate, and provide written informed consent. Capacity will be assessed and consent taken by a research assistant.

Exclusion criteria for patient participants include comorbidity preventing participation (e.g. severe breathlessness, pain, psychosis, Parkinson’s disease, or other severe neurological disease), life expectancy of less than 1 year, or being unlikely to undertake the intervention regularly (e.g. planned elective surgery, planning to move away, or commitments elsewhere).

Individual participants will discontinue participation if: they withdraw consent or no longer wish to take part; the therapist overseeing their care decides the participant is no longer able to take part (for example due to inter-current illness or injury, progression of their disease or inability to adhere despite adjustment and tailoring of the programme); otherwise at the discretion of the investigator (e.g. risk to safety of staff). Participants may opt not to take part in individual therapy sessions or a series of sessions (e.g. because of holidays or intercurrent illness) without withdrawing from the study. We will collect outcome data from those who withdraw if they are willing.

No formal sample size was calculated for the feasibility study; however, a sample size of 60 was considered sufficient to answer feasibility questions and enable the conduct of the adherence and process of evaluations.

Carer participants will be partners, family members or others in a caring relationship, who are in contact with the patient participants most weeks, are willing to take part and can communicate in English.

For the ‘optimising engagement and adherence’ and process evaluations, we will conduct interviews and focus groups. We will undertake three small focus groups with patients with mild dementia, and their family carers, each comprising three to five participants to investigate promotion of adherence. In the process evaluation, we will conduct semi-structured interviews with about ten participants in each active-treatment arm (moderate- and high-intensity supervision). Carers will be interviewed separately or together with the participant, depending on their preference. We will select for interview participants with low- and high-adherence, identified through their exercise diaries, self-report or therapist-report. We will also seek to interview a sample of participants who discontinue the intervention (up to 10, and their carers). We will undertake two to three staff focus group discussions, each of six to eight members.

### Randomisation

Participants will be individually randomised on a 1:1:1 ratio, stratified by site, co-resident carer and history of falls, using an independent, secure, web-based, randomisation procedure that can be accessed 24 h a day and held at the NWORTH clinical trials unit, Bangor University [78]. The randomisation system will be maintained by a statistician independent of the analysis and research teams to ensure blinding of allocation and analysis.

### Blinding

A research assistant will perform the randomisation and communicate the allocation to the intervention service provider. Research assistants collecting baseline and follow-up data by questionnaires or telephone will be blind to treatment allocation, and will request participants not to reveal their treatment group. Due to the nature of the intervention, blinding of the intervention is impossible for participants and therapists administering it.

### Baseline data

Pairs of research assistants will administer data collection questionnaires by interviewing participant and carer separately at the participant’s home.

Baseline data will comprise:

- Demographic and contact details for patient and carer participants
- Medical and falls history, including previous fractures, recent hospitalisation and medication
- Falls risk factors, including vision and lying and standing blood pressure
- Gross cognition (sMMSE [76], Clinical Dementia Rating [79])
- CANTAB neuropsychological assessment: Paired Associated Learning (PAL), Attention Switching Task (AST), Spatial Working Memory (SWM) (Cambridge Cognition, Cambridge, UK)
- Verbal fluency (from MOCA test [75])
- Scaled outcome variables measuring activities of daily living (ADL) (Disability Assessment for Dementia scale, DAD [80], Nottingham Extended ADL Scale [81]), activity (Incidental and Planned Activity Questionnaire, IPAQ) [82], quality of life (EQ-5D [83], DEMQoL (participant and proxy) [84]), fear of falling (short falls efficacy scale, FES-I [85]), Hospital Anxiety and Depression Scale (HADS) [86], muscle strength (Lafayette dynamometer, Lafayette, IN), Berg Balance Scale [87], Timed Up and Go test [88], SHARE frailty instrument [89].
- Carer strain (caregiver strain index) [90].
- Participant and carer service use (Client Service Receipt Inventory, CSRI) [91].

### Intervention

The intervention was developed by a multi-disciplinary team of physiotherapists, occupational therapists, doctors, nurses, psychologists, and patient and public representatives, using the principles of co-production. This was informed by findings from three literature reviews [92–94], clinician, health psychology and neuropsychological expertise ascertained by interview and during workshops, focus group discussions held with patients, carers, and clinicians, and the views of patients and their carers from a prior interview study [62]. Ten patients were treated in a 6-week ‘proof of concept’ study as part of the intervention development, in which intermediate outcomes were measured and field notes were thematically analysed. A comprehensive manual was written, and a training programme devised for physiotherapist, occupational therapist and rehabilitation support worker staff. A therapy workbook was designed to be left in participants’ homes.

The intervention is grounded in established fall prevention expertise, the Assessment of Motor Process Skills activity assessment [95] and theories of motivation (e.g. self-determination theory). It includes a professional assessment of ability, risk and goals. We will use capability-based ‘tailoring’: using assessed neuropsychological impairments, usual level of physical activity, co-morbidities, aligning activities to interests, and assessing priorities through goal-identification and goal-setting. A programme of activities and exercises will be agreed with the patient and carer. Therapy includes functional activity, training and advice, environmental assessment, strength and balance exercises, and dual-task training. The programme will be set out in the participant’s workbook in a format easily accessible to them and their carer. Participants will be encouraged to perform exercises three times per week, and partners, family members or carers will be asked to prompt and support, by telephone if necessary, or to participate as well. The programme will be progressive, intensive and supported by varying intensity of supervision from the research therapists (Fig. 1).

The control arm will be the offer of a standard falls assessment and advice completed by a therapist, with up to two follow-up visits if thought clinically necessary.

The moderate-intensity supervision arm participants (3-month programme) will receive a total of 11 treatment sessions: 6 from an occupational therapist (OT) and 5 from a physiotherapist over 12 weeks. Participants will be expected to exercise and complete activities independently between supervised sessions, and taught to continue after the supervision period has stopped. The moderate-intensity intervention was modelled on the intervention in an ongoing Australian trial [96, 97].

The higher-intensity supervision arm (12-month programme) will comprise 11 treatment sessions from registered therapists: 6 from an occupational therapist, 5 from a physiotherapist plus supervised support from a rehabilitation support worker (RSW) twice a week for 3 months, once a week for 3 months, once a fortnight for 3 months, and once a month for 3 months. In total they will get 51 treatment sessions, and will be asked to continue with the intervention programme independently as supervision tapers. At initial, review and progression points, the RSW will visit jointly with the therapists.

The feasibility and practicability study, and associated process evaluation, forms part of the development process for the intervention, which will be subject to further refinement based on the findings. For this reason, we will not publish the manual until the intervention is finalised (anticipated September 2018).

### Follow-up

Researchers will visit participants at home at 12 months (± 2 weeks) to complete health status assessments by interview with the participant and informant. Participants will keep a daily diary for 12 months, detailing activities and exercises undertaken and recording falls. This will be supported and prompted by monthly telephone calls [98]. Measures will be taken to ensure that falls ascertainment is not biased across the intervention arms (e.g. therapists or RSWs will be asked not to prompt recall of falls). Health and social care use will be ascertained during the telephone calls, using electronic healthcare records and the Client Service Receipt Inventory (CSRI) at follow-up interview. Persistence with the therapy programme, health and social care use, and hospitalisation will be ascertained by short questionnaire at 18 and 24 months.

### Outcome measures

#### Trial feasibility outcomes

The feasibility outcomes are recruitment, retention, adherence and acceptability of the intervention, and completion of baseline and outcome data. A recruitment log will be maintained, and recruitment and withdrawal rates recorded.

#### Health status outcome measures

- Disability Assessment for Dementia (DAD) ADL scale [80]
- Nottingham Extended ADL scale [81]
- IPAQ activity questionnaire [82]
- CANTAB neuropsychological assessment: Paired Associated Learning (PAL), Attention Switching Task (AST), Spatial Working Memory (SWM)
- Verbal fluency
- DEMQoL and EQ-5D quality of life questionnaires [83, 84]
- Short falls efficacy scale (fear of falling, FES-I) [85]
- Hospital Anxiety and Dementia Scale (HADS) [86]
- Berg balance Scale [87]
- Single- and dual-task Timed up and Go (TUG) test [88]
- SHARE frailty instrument [89]
- Muscle strength (Lafayette dynamometer)
- Resting and post-exercise pulse rate.
- Carer strain [90]

#### Falls and activity

Study-related and other activity will be quantified by time using the daily diary. Falls and injurious falls will be counted. Participants will be asked to wear pedometers in weeks 1, 26 and 50 as an objective measure of activity.

#### Process evaluation, motivation and adherence study

We will record the number of therapy sessions delivered. We will estimate amount of exercises and prescribed activities done independently using the daily diary.

Data on habit formation will be collected at alternate months by telephone, using a standardised scale, the Self-Reported Habit Index [99].

At months 1, 3 and 6 data on participant’s and staff perceptions of motivational communication will be collected by telephone, using the Healthcare Climate Questionnaire, adapted for exercise settings [100, 101].

Central to self-determination theory is the concept of basic psychological needs [102]. These needs (for competence, autonomy, and relatedness) must be satisfied for people to develop and function in healthy or optimal ways [103]. We will record psychological variables hypothesised to be related to adherence at baseline and follow-up:

- Behavioural Regulation in Exercise Questionnaire (BREQ-2) [104]
- Basic Psychological Need Satisfaction and Frustration Scale [105] (participants and staff)
- Clinician’s work motivation (Work Extrinsic and Intrinsic Motivation Scale) [106].

Qualitative interviews will take place at home. Therapists and their assistants delivering the intervention will take part in focus group discussions, with an experienced facilitator, held at a convenient location. Interviews and focus group discussions will be audio-recorded. We will ask about: the experience of undertaking the programme; perceived benefits or value; difficulties, barriers, and facilitators; mechanisms of behaviour change and habituation to the exercise regime; the impact of supervision intensity and other factors on acceptability and adherence; and whether level of supervision can be matched to individual participant characteristics; reasons for high- or low-levels of adherence, or discontinuation of the programme; the acceptability of carer support; and the extent of burden this might impose.

Thirty purposively-selected therapy sessions will be video-recorded, to include a range of clinicians and stages of the intervention.

The experience of delivering the intervention will be explored in focus groups with staff. We will discuss: adequacy of the training; the experience of delivering the intervention; perceptions of patient and carer responses; perceived effectiveness; barriers to implementation and how these may be overcome.

### Adverse events

This is a non-drug intervention trial, using interventions that are within current recommended exercise guidelines, and might be offered as part of a routine clinical service. As such, the risk of severe or unexpected adverse events is low. Dementia is progressive and both cognition and function may deteriorate in the course of the study. Comorbidity and inter-current illness will be very common. A balance must be drawn between ensuring the safety of participants, and failing to identify specific risks amidst numerous reports of un-related incidents.

Falls, injuries, hospital admissions and deaths will be ascertained prospectively as part of the trial outcomes. They will be recorded through diaries, supported by telephone calls from (blinded) researchers where necessary, and by examining electronic health records.

We will define an adverse event as an incident, injury or symptom related to therapy sessions, or exercise undertaken independently. The most likely adverse events are fatigue, minor musculo-skeletal symptoms or injuries such as muscle stiffness, or sprains, or increased falls though increased activity. Some conditions such as arthritis or angina may be exacerbated by exercise. Adverse events will be monitored by therapists and RSWs, and reported where they occur. However, this will give a biased impression of prevalence, as the different treatment arms have substantially different amounts of contact with professional staff. Medical judgement will be exercised in deciding whether an adverse event is serious, expected or causally-related.

### Data management

Data management will be according to the Standard Operating Procedures of NWORTH CTU. Quantitative data will be entered into a MACRO database, written and maintained by NWORTH CTU, by research assistants. This incorporates range checks at the point of data entry. Data will be further checked by the study statistician, and a sample verified against source documents. Qualitative (audio-and video-recorded) data will be transcribed and anonymised, and managed using NVivo 11 software (QSR International, Daresbury, UK). The research grant co-applicants will have access to the final dataset. Paper records will be securely archived according to University of Nottingham standard operating procedures.

### Statistics and data analysis

#### Feasibility analysis

The main analysis will be of feasibility outcomes. We will calculate:

- Recruitment rate (randomisations per month)
- Retention rate (proportion completing therapy programme and study follow-up)
- Therapy adherence rate (proportion of professionally-supervised visits undertaken, and time spent doing self-directed therapeutic exercise or activity)
- Missing data (proportion fully completed, for each scale, at each time point).

Participant throughput and flow will be summarised for each trial arm using a CONSORT diagram. Reasons for non-eligibility, non-treatment, withdrawals and non-completion of questionnaires will be reported.

#### Efficacy analysis

Descriptive statistics (e.g. means, medians, proportions, standard deviations and ranges) of baseline demographic, health status and service use data will be calculated for each trial treatment arm.

We will compare changes in health status outcomes between the three trial arms.

The Disability Assessment for Dementia and Nottingham Extended ADL scale outcomes will be compared using an analysis of covariance to adjust for the baseline score and stratifying variables. The nature of the distribution of the falls data will also be examined to determine how best to analyse the falls data in the main trial. Statistical analysis will be performed on an intention-to-treat basis. There will be no imputing of missing data. Assumptions in determining the sample size of the main trial will be checked.

#### Qualitative analysis

Data will be coded and a thematic analysis undertaken using the principles of constant comparison [107, 108]. A separate coding frame will be developed for each dataset after which themes from each will be systematically compared. Each coding frame will be developed through the independent coding of about five transcripts and associated field notes by at least three researchers. Iterative comparison and discussion will continue until the coding frame has stabilised. Subsequent transcripts will be coded by a research fellow, with 10% double-coded to establish consistency and increase comprehensiveness. Coding will incorporate a priori topics of relevance to the study; for example: ‘understanding of dementia’, ‘relevance of exercise’, ‘motivation’, ‘burden’, ‘comorbidity’ and others identified inductively from the data.

Coding proceeds through three stages. Open coding (indexing of sections of text/video themes to ‘nodes’); detailed scrutiny of the content of each node (‘coding on’) when coding is refined through reorganisation and reallocation into subnodes, and regrouping of nodes and subnodes into a hierarchical structure (‘tree’); relating sub-nodes to overarching core themes. Each data set will be subject to both separate and integrated analysis. Findings will be synthesised through charting and matrix displays.

#### Process evaluation

We will follow MRC guidance (2014) [73] on process evaluation, which describes three components using a mixed-methods approach: implementation or delivery; mechanisms of impact; contextual factors.

Implementation (delivery of intervention), includes fidelity (quality of delivery) and dose (quantity of delivery). Records of therapy sessions undertaken and self-directed activity will be examined, and video-recorded therapy sessions will be assessed qualitatively for fidelity. Mechanisms of impact and contextual factors include engagement and adherence. These will be investigated through the qualitative studies.

We will also do a qualitative analysis of 10 purposively-selected video-recorded intervention sessions, which will be transcribed and analysed. Sessions will be selected to include a range of professionals, patient participants and stages of intervention delivery. We will use these to derive insight into: intervention delivery in real world settings; its consistency; interactions between patient, carer and professional participants; patient and carer responses and engagement with the intervention; the circumstances and contextual factors influencing engagement; models of best practice in delivering the intervention.

#### Optimising uptake and adherence

Using the Capability, Opportunity, Motivation – Behaviour (COM-B) framework [58], we will seek evidence on what support participants would like, or will need, to achieve short- and long-term adherence. We will review methods to support uptake and adherence in exercise interventions reported in the literature, drawn from a variety of conditions, which might be adapted or used for people with dementia. We will develop a strategy for promoting and supporting uptake, adherence and persistence with the intervention, develop guidance on tailoring, need for direct supervision, and describe habit formation and factors that predict successful adherence.

We will analyse baseline distributions, changes and associations with psychological variables hypothesised to determine motivation and adherence, including motivation of participants to exercise (BREQ-2 [102]), and basic psychological needs, [105], and clinician factors, including communication style [100]), work motivation [106], and basic psychological needs [105].

#### Economic analysis

Preliminary cost-effectiveness analysis will be undertaken from a National Health Service and personal social services (public sector multi-agency) perspective with short-term time horizon relating to the trial follow-up period. Cost-effectiveness analysis will be conducted in line with NICE guidelines for technical appraisal [109] and MRC guidelines for the evaluation of complex interventions [110]. A primary cost-effectiveness analysis will be undertaken on cost per point improvement on the Disability Assessment for Dementia scale. A secondary cost-effectiveness analysis will be undertaken on the cost per fall averted. A cost-utility analysis will be undertaken using EQ-5D-3 L and DEMQoL as sources of utility weights for calculating quality adjusted life years (QALYs) [111, 112]. Data from the feasibility study will be used to model longer-term costs and benefits to estimate potential lifetime savings to the NHS and social care of an observed improvement in ADL. Deterministic and probabilistic sensitivity analysis will be undertaken. Social Return on Investment (SROI) analysis will be undertaken and a SROI ratio will be generated from the total value of inputs and total value of outputs [113, 114]. The resulting ratio will be the amount of social value generated for every £1 invested in the programme.

Sensitivity analysis will be conducted to vary the costs of inputs (e.g. the cost of the staff delivering the programme), and to vary the discount rate applied (base case rate of 3.5%, sensitivity analysis rate of 1.5%) in accordance with NICE guidelines [115].

### Governance

An independent Programme Steering Committee and Data Monitoring Committee have been constituted, each with an independent chair, expert clinician, statistician and (on the steering committee) patient and public contributor. The Trial sponsor is Nottingham University Hospitals NHS Trust.

## Discussion

There is a pressing need for interventions to maintain activity and independence among people with dementia. This is to enable people to ‘live well’, to help prevent crises, and to mitigate the increasing burden on health and social care of dementia-related dependency. Intervening at an early stage may enable people to establish new health habits before the inevitable progression of dementia. Fall-related injury and activity restriction are often responsible for deterioration, or a ‘spiral of decline’. Falls prevention for older people in general is well-established, but this has not yet been adapted for people with dementia.

We present the protocol of a complex study, aiming both to establish the feasibility of conducting a large RCT of an exercise intervention for people with early dementia, and to optimise implementation of the intervention so that the RCT will evaluate an intervention that is practical, well-justified and suitable for wider-scale subsequent adoption.

The development and evaluation of complex interventions is a methodological challenge [73, 110]. Our stance is that if an intervention is suitable for testing on a randomised basis, then an RCT is the best method to use. Other methodologies, such as realist evaluation, may also contribute to understanding complex interventions. The embedding of a process evaluation and work to study engagement and adherence, will allow us to answer questions about ‘what works, for whom, under what circumstances and why?’ [116].

Several conditions need to be met for an RCT to be successful. In common with all RCTs, we must establish research feasibility: recruitment rates, data collection, follow-up and the properties of outcome measures. But for complex interventions, it is also necessary that the intervention tested is deliverable and optimised. RCTs are expensive and labour-intensive; adequate preparatory work is required to ensure that the intervention is fit for testing, and sufficiently well-defined to be repeatable. For applied health research that anticipates early patient benefit, the intervention should be suitable for widespread adoption in health care services if the results are favourable.

Our study is designed to examine whether these conditions are satisfied, and to enable adjustment or adaptation in the light of experience. Our intervention is based upon work we have conducted over several years examining, and contributing towards, a wide body of physiological evidence that links exercise to improved outcomes [19, 55, 57, 92–94]. The feasibility RCT examines two variants of the exercise intervention with higher and lower degrees of supervision, on the basis that a lower intensity support intervention would be less expensive to deliver, but might not ensure the long-term adherence presumed necessary for benefits to be seen, whilst more intensive supervision will be more expensive but may be more effective [34–36, 48, 57]. The decision to choose one arm or the other, or how to tailor supervision according to individual circumstances, in a subsequent large RCT will depend upon many factors, critically including the degree to which long-term adherence is established. This will be informed by the process evaluation, and motivation and adherence study. Given that both intervention arms will incur health care costs, it is necessary to demonstrate whether there is a reasonable likelihood of health gains before conducting a large and expensive RCT, and any subsequent health benefit justifies the costs of the intervention. Economic appraisal towards the end-of-life is contentious, ideally requiring an approach which can model costs and benefits over the whole remaining lifespan, and adopting a wider frame of reference than cost per QALY alone. For this reason we are undertaking preliminary economic analyses, including Markov modelling and social return on investment analyses.

We face several methodological challenges. Research involving people with dementia can be difficult, even when impairments are mild. However, we have expertise in doing such studies. We note that people with dementia and their families are keen to be involved in research. The provisions of the English Mental Capacity Act are designed to enable inclusion, and our governance processes ensure that studies are conducted ethically. Many studies have demonstrated that people with dementia can be successfully recruited to trials, and useful and valid patient-centred outcomes can be measured. The recording of falls and levels of activity will be challenging, and will be examined in this study.

The studies described represent part of a research programme aimed at understanding activity-limitation among people with dementia, how to intervene to promote activity and reduce falls, and how to get such a therapy programme to work in practice. The protocol describes work on the final stages of developing the intervention, preparing a definite RCT, and data for economic modelling.

The definitive RCT is due to recruit from September 2018 and complete in 2021.

## Abbreviations

ACE-IIII: Addenbrooke’s Cognitive Assessment
ADL: Activities of daily living
AMPS: Assessment of motor process skills
BREQ-2: Behavioural Regulation in Exercise Questionnaire
CSRI: Client service receipt inventory
FES-I: Falls efficacy scale international
HADS: Hospital Anxiety and Depression Scale
IPAQ: Incidental and Planned Activity Questionnaire
MCI: Mild cognitive impairment
MoCA: Montreal Cognitive Assessment
NWORTH: North Wales Organisation for Randomised Trials in Health
PrAISED: Promoting Activity, Independence and Stability in Early Dementia.
RCT: Randomised controlled trial
RSW: Rehabilitation support worker (unregistered therapy assistant)
SHARE: Survey of Health Ageing and Retirement in Europe
sMMSE: Standardised mini-mental state examination
